# Extracellular vesicles from equine mesenchymal stem cells decrease inflammation markers in chondrocytes in vitro

**DOI:** 10.1111/evj.13537

**Published:** 2021-11-24

**Authors:** Magdalena Arévalo‐Turrubiarte, Mario Baratta, Giovanna Ponti, Elisabetta Chiaradia, Eugenio Martignani

**Affiliations:** ^1^ Department of Veterinary Science University of Turin Turin Italy; ^2^ Department of Chemistry Life Sciences and Environmental Sustainability University of Parma Parma Italy; ^3^ Department of Veterinary Medicine University of Perugia Perugia Italy

**Keywords:** chondrocytes, extracellular vesicles, horse, mesenchymal stem cells, pro‐inflammation

## Abstract

**Background:**

Mesenchymal stem cells (MSCs) have been used therapeutically in equine medicine. MSCs release extracellular vesicles (EVs), which affect cell processes by inhibiting cell apoptosis and regulating inflammation. To date, little is known about equine EVs and their regenerative properties.

**Objectives:**

To characterise equine MSC‐derived extracellular vesicles (EVs) and evaluate their effect on equine chondrocytes treated with pro‐inflammatory cytokines in vitro.

**Study design:**

In vitro experiments with randomised complete block design.

**Methods:**

Mesenchymal stem cells from bone marrow, adipose tissue, and synovial fluid were cultured in vitro. The MSC culture medium was centrifuged and filtered. Isolated particles were analysed for size and concentration (total number of particles per mL). Transmission electron microscopy analysis was performed to evaluate the morphology and CD9 expression of the particles. Chondrocytes from healthy equines were treated with the inflammatory cytokines interleukin (IL)‐1β and tumour necrosis factor‐alpha. MSC‐derived EVs from bone marrow and synovial fluid cells were added as co‐treatments in vitro. Gene expression analysis by real‐time PCR was performed to evaluate the effects of EVs.

**Results:**

The particles isolated from MSCs derived from different tissues did not differ significantly in size and concentration. The particles had a round‐like shape and positively expressed CD9. EVs from bone marrow cells displayed reduced expression of metalloproteinase‐13.

**Main limitations:**

Sample size and characterisation of the content of EVs.

**Conclusions:**

EVs isolated from equine bone marrow MSCs reduced metalloproteinase 13 gene expression; this gene encodes an enzyme related to cartilage degradation in inflamed chondrocytes in vitro. EVs derived from MSCs can reduce inflammation and could potentially be used as an adjuvant treatment to improve tissue and cartilage repair in the articular pathologies.

## 1 INTRODUCTION

Osteoarthritis (OA) and tendonitis limit motion and performance of race and sport horses.^1^, ^2^ Mesenchymal stem cells (MSCs) have been used for treatment in equine OA and tendonitis^1^, ^3^ and isolation of MSCs from bone marrow (BM), adipose tissue, umbilical cord, and synovial fluid has been described.^1^, ^3^ BM cells implanted in injured tendons can improve the morphological orientation of the affected fibres.^1^ In addition, MSCs have anti‐inflammatory and immunomodulatory properties.^3^ MSCs can regulate the production of tumour necrosis factor‐alpha (TNF‐α) to mediate inflammation and suppress T‐cell proliferation.^4^ Paracrine mechanisms in MSCs are important for the activation of their immunomodulatory potential.

When MSCs are exposed to an inflammatory environment, inflammatory inhibitors are released.^4^ MSCs also release vesicles of different sizes, collectively known as extracellular vesicles (EVs).^5^ EVs are classified and named according to their size and release mechanisms.^6^ In general, exosomes are secreted by exocytosis and range in size from 30 to 120 nm, while shedding vesicles, also known as microvesicles, are released via budding from the plasma membrane. Microvesicles range in size from 80 to 1000 nm.^5^, ^6^ EVs have various functions, including tissue regeneration properties by limiting tissue injury, participating in regenerative processes,^5^ and enhancing cell proliferation and apoptosis.^7^ EVs also carry proteins and genetic information in the form of mRNA and microRNA and participate in cell‐to‐cell communication^6^ by interacting and fusing with the lipid membranes of target cells, allowing EVs to deliver proteins and genetic information.^5^

Extracellular vesicles have also been suggested as a potentially potent treatment for tendon tissue repair in equines,^5^ and other studies have demonstrated the performance of EVs in OA. Improvement of cell proliferation in chondrocytes and osteogenesis in bone has been reported after administration of EVs derived from MSCs in vivo.^8^ EVs have an anti‐inflammatory effect in chondrocytes derived from human OA patients in vitro.^9^ In addition, EVs participate in the decrease of pro‐inflammatory ILs,^9^ reduce T and B lymphocyte proliferation in vitro, and improve arthritic mouse inflammation in vivo.^10^

Few studies have been performed on EVs derived from equine MSCs. Transmission electron microscopy (TEM) of EVs derived from equine adipose cells showed rounded shapes of different sizes.^11^ Another study characterised EVs from adipose cells by their expression of CD9, CD63, and CD81 surface markers.^12^ However, little is known about EVs derived from other types of MSCs in horses, and their effect on inflammation remains unclear. Since MSCs from BM, adipose, and synovial fluid (SF) cells have been proposed as a therapeutic option for joints in equines,^1^ the objective of our study was to isolate and evaluate EVs derived from different cell types (BM‐MSCs, SF‐MSCs, and adipose MSCs) and study the effect of BM‐ and SF‐MSC derived EVs in a pro‐inflammatory experiment in vitro using equine primary chondrocytes. These findings could indicate the potential therapeutic value for tissue treatment and MSC‐derived EVs.

## 2 MATERIALS AND METHODS

### 2.1 Culture of mesenchymal stem cells

Mesenchymal stem cells derived from BM, SF, and adipose cells from the mesenteric, tail, and neck fat (NF) were isolated and cultured as described previously.^13^ All cell types were isolated from three male horses 10‐16 months of age. The samples were collected at a local slaughterhouse, according to the Italian regulations under the supervision of the Veterinary Services of the Italian National Health Service, Ministry of Health. MSCs were seeded in 60 mm tissue culture (TC) dishes (Sarstedt) and cultured until they reached 80% confluence. Cells were washed with phosphate‐buffered saline (PBS), and the medium was replaced to prepare for EV collection. The proliferation medium consisted of 1× Dulbecco's Modified Eagle's Medium (DMEM) with 4.5 g/L D‐glucose, 2 mmol/L L‐glutamine, 2.5 µg/mL amphotericin B, 1% penicillin‐streptomycin (all from Sigma‐Aldrich), and 10% of EV‐free foetal bovine serum (FBS; Gibco). The EV‐free FBS was made by ultracentrifuging commercially available FBS at high speed (100 000 *g*) at 4°C overnight (18 hours) using an L8‐70M L8M class 7 ultracentrifuge with a SW4ITI swinging bucket rotor (both from Beckman‐Coulter) and then filtering the solution with a 0.2 µm syringe filter.^14^

### 2.2 Isolation of extracellular vesicles

Approximately 5‐15 mL of medium was recovered after 24 hours of MSCs culture. The dish was washed with 5 mL of PBS and was added to the collected medium. Samples were centrifuged at 259 *g* for 5 minutes at 4°C in a refrigerated centrifuge (Eppendorf 5804R equipped with a model A‐4‐44 rotor, both from Eppendorf). The supernatant was transferred to a clean tube and centrifuged at 1560 *g* for 5 minutes at 4°C. The collected supernatant was filtered (0.2 µm) and placed in polyallomer conical tubes (Beckman‐Coulter). Ultracentrifugation at 100 000 g was performed for 1 hour at 4°C with a no‐break deceleration. The resulting pellet was recovered in 500 µL of PBS (Sigma‐Aldrich) for further analysis. Frozen samples were stored at −80°C in 1% dimethyl sulfoxide (DMSO, Sigma‐Aldrich).

### 2.3 Nanoparticle analysis

The EV samples were diluted (1:200) before acquisition with PBS. They were analysed using a NanoSight LM10 particle size analyser (NanoSight Ltd.) equipped with a 405 nm laser and a microscope with a Hamamatsu digital camera C11440 (ORCA Flash 2.8) at 20× magnification. Particle concentration and size were determined three times during a 30 seconds video. Data were generated by NTA 3.1 nanoparticle tracking analysis software. Three technical replicates were analysed for each horse sample (n = 3 biological replicates).

To calculate the yield, the number of EVs from the nanoparticle analysis was multiplied by the dilution factor used for sample preparation (200×) and divided by the volume of the MSCs culture medium recovered after 24 hours. Therefore, the yield represents the number of EVs produced by MSCs per ml of culture medium over a 24 hours time period.

### 2.4 Transmission electron microscopy

Isolated EVs were placed on coated grids (Pioloform^®^ film 200 copper mesh, Agar Scientific) and fixed with 1.25% glutaraldehyde in 1% paraformaldehyde in 0.1 M Sorensen's phosphate buffer for 15 minutes. Each grid was washed five times for 10 minutes each time using Sorensen's phosphate buffer. Excess liquid was removed using Whatman^®^ filter paper. The grids were dried at room temperature prior to analysis. Immunogold staining consisted of washing with Tris‐buffered saline (TBS), followed by incubation with goat serum (10%) and an immunogold buffer for 1 hour. Subsequently, a two‐night incubation with the primary antibody CD9 (1:10, Clone HI9a, anti‐mouse, BioLegend) at room temperature was performed, followed by a 1‐hour incubation with anti‐mouse IgG immunogold secondary antibody (EM.GAM10, BBI Solutions). Three 15 minutes washes with PBS were then performed, followed by fixation in 2.5% glutaraldehyde for 10 minutes. Three more 15‐minutes washes were performed on the samples using PBS, followed by overnight drying at room temperature before image acquisition. A JE1‐1010 transmission electronic microscope (JEOL) was used to observe the grids at 250 000× magnification. Image acquisition was performed using soft imaging system analysis® (Megaview III soft imaging system).

### 2.5 Pro‐inflammatory assay

Chondrocytes were isolated from the healthy metacarpal/metatarsophalangeal joint of three young horses (5‐10 years of age).^15^ Chondrocytes used in the assay were from early passages (passages one to three). EVs were previously isolated from MSCs cultured in a 150 mm dish (CytoOne, Starlab), as described in the *Isolation of* extracelluar vesicles section 2.2. To test the effect of cytokines on the proliferation of equine chondrocytes, 300 000 chondrocytes were seeded in each well of a 6‐well plate (CytoOne, Starlab). Cells were starved with FBS‐free medium (DMEM supplemented with 2% bovine serum albumin [BSA], all from Sigma‐Aldrich) for 24 hours. TNF‐α or IL‐1β was then added to the culture medium at two different concentrations (10 or 50 ng/mL). Three technical replicates were prepared for each treatment. After 24 hours, the culture medium was removed, and cells were washed once with PBS. Fresh PBS supplemented with 1 mg/mL Hoechst 33342 (Thermo Fisher Scientific) was added to each well to stain the nuclei. Images of ten random fields per well were acquired at 40× magnification using a Leica AF6000 LX inverted microscope equipped with a Leica DFC350 FX monochrome digital camera (Leica Microsystem). Nuclei were then automatically counted using ImageJ software (version 1.52s; https://imagej.nih.gov/ij/). For the pro‐inflammatory assay, 300 000 equine chondrocytes were seeded in individual wells of a 6‐well plate with proliferation medium (1× DMEM + 10% EV‐free FBS, 4.5 g/L D‐glucose, 2 mmol/L L‐glutamine, 2.5 μg/mL amphotericin B, and 1% penicillin‐streptomycin; all from Sigma‐Aldrich). Chondrocytes were starved for 24 hours with DMEM containing 2% BSA. Treatments were initiated and consisted of 10 ng/mL of IL‐1β, 10 ng/mL of TNFα, and each cytokine with purified EVs (13 333 EVs/cell, adapted from Collino et al^7^) derived from the different types of MSCs. Chondrocytes were then processed for RNA extraction and gene expression analysis. Each treatment was performed in two technical replicates using EVs derived from two types of MSCs (three biological replicates for BM‐ and SF‐MSC derived EVs).

### 2.6 Gene expression

According to the manufacturer's protocol, chondrocytes were lysed, and total RNA was extracted using TRI Reagent (Sigma‐Aldrich). The resulting RNA was eluted with 20 µL diethylpyrocarbonate (DEPC; Sigma‐Aldrich) purified water and analysed using a NanoDrop 2000 spectrophotometer and NanoDrop software (ThermoFisher Scientific). Stable RNA was used for reverse transcription‐polymerase chain reaction (RT‐PCR) using an iScript™ kit (Bio‐Rad). Samples were processed using Bio‐Rad iCycler. Real‐time PCR was carried out using SsoAdvanced Universal SYBR Green Supermix (Bio‐Rad) and run in a Bio‐Rad CFX real‐time system. Real‐time PCR products were obtained following these conditions: initial denaturation at 95°C for 3 minutes and 40 amplification cycles (95°C for 10 seconds, then 62°C for 30 seconds, 95°C for 10 seconds), and a melting curve starting at 65°C with an increment of 0.5°C for 5 seconds. Primers used for this study were verified using the nucleotide basic local alignment search tool (BLAST, National Center for Biotechnology Information, National Library of Medicine, National Institutes of Health, Bethesda, MD, USA) and the Eurofins Oligo Analysis Tool (www.eurofinsgenomics.eu) searching for equine‐specific sequences (*Equus caballus*) (Table 1). IL‐6, a disintegrin and metalloproteinase with thrombospondin motif 4 (ADAMTS4), ADAMTS5, tissue inhibitor of metalloproteinase 3 (TIMP3), matrix metalloproteinase‐2 (MMP‐2), and transcription factor SOX‐9 primers were designed using the tools mentioned above. TIMP 1 and 2,^16^ MMP‐3,^17^ MMP‐13,^18^ aggrecan (ACAN), collagen type II (COL2),^19^ and the housekeeping gene hypoxanthine phosphoribosyl‐transferase 1 (HPRT) were used as described previously. Quantitative data were obtained using Bio‐Rad CFX Maestro 1.1 software. Relative gene expression was normalised to HPRT and calculated using the 2^ΔΔCT^ Livak method.

**TABLE 1:** Primer sequences used for gene expression in the pro‐inflammatory experiment

Gene	Forward	Reverse
IL‐6	GGCTACTGCTTTCCCCACC	CCCAGATTGGAAGCATCCGT
ADAMTS‐4	CATGTGCAACGTCAAGGCTC	AGTCACCACCAAGCTGACA
ADAMTS‐5	GAGATGACCATGAGGAGCACTAC	GGCCATCGTCTTCAATCACAG
TIMP‐1	ATCCCCTGCAAACTGCAGAGT	GCCCTTGTCAGAGCCTGTGA
TIMP‐2	AGAGTTGTTGAAAGTCGACAAGCA	ACCGAGCGATCACTCAGGAA
TIMP‐3	GACGCCTTCTGCAACTCTGA	GTACTGCACATGGGGCATCT
MMP‐1	AGGAGCCCAGTCGTTGAAAA	GTTCCCTTCGGTGAGGACAA
MMP‐2	TGAGCTCCCGGAAAAGATCG	AAAGGCAGCATCCACTCGTT
MMP‐3	GCAAGGGACGAGGATAGCAA	GTCTCATTTCTTTTCCAAGGTCGTAGT
MMP‐13	ACAAGCAGTTCCAAAGGCTAC	CTCGAAGACTGGTGATGGCA
ACAN	ACAACAATGCCCAAGACTAC	GCCAGTTGTCAAATTGCAAG
Sox9	ACGCCGAGCTCAGCAAGA	CGCTTCTCGCTCTCGTTCA
COL2	TGAAACTCTGC CACCCTGAATG	TTGTCCTTGCTCTTGCTGCTC
HPRT	TGA CAC TGG CAA AAC AAT GCA	GGTCCTTTTCACCAGCAAGCT

### 2.7 Data analyses

Data collected using NanoSight were analysed. Differences in the size and concentration of isolated particles were evaluated using the Kruskal–Wallis test. Differences were considered statistically significant at *P* < .05. Results derived from the real‐time PCR analysis were evaluated first using a Shapiro‐Wilk test to evaluate normality in the data. Normally distributed samples were evaluated using a two‐way ANOVA. Samples that were not normal were analysed using a non‐parametric Kruskal–Wallis test. If the data were statistically significant (*P* < .05), a *post hoc* test was used for both tests using a Bonferroni *post hoc* test and non‐parametric Wilcoxon‐Mann‐Whitney analysis. Data are reported as means of fold‐average change. Statistical analyses were performed using IBM SPSS software version 25.

## 3 RESULTS

### 3.1 Isolation of extracellular vesicles from different types of mesenchymal stem cells

Collection of media from derived MSCs was performed after 24 hours in culture with EV‐free FBS. Ultracentrifugation of this media from derived MSCs cultures served to recover EVs, and nanoparticle tracking analysis was performed. The size of the particles was the expected size for EVs.^6^ The concentration of particles derived from each type of isolated MSCs (BM, SF, NF, mesenteric, and tail fat cells) and size distribution was analysed. No significant differences (*P* > .05) were observed in the concentration of all samples (Table 2).

**TABLE 2:** Overview of nanoparticle tracking analysis from MSCs‐derived media

Cell type	Concentration of EVs (particles/mL)	Size (nm)
Bone marrow	1.87 ± 8.41 × 10^10^	235.9 ± 40.50
Synovial fluid	1.69 ± 2.47 × 10^11^	180.2 ± 64.45
Neck fat	3.88 ± 1.60 × 10^10^	151.2 ± 36.27
Mesenteric fat	2.43 ± 1.98 × 10^10^	217.8 ± 8.28
Tail fat	5.85 ± 4.36 × 10^10^	153.8 ± 62.24

Particles from BM cells seemed to be larger in comparison with other MSCs types. NF particles were smallest. No significant differences were observed (*p* > 0.05). Mean data (n = 3) are presented as average size ± SD.

**FIGURE 1:**
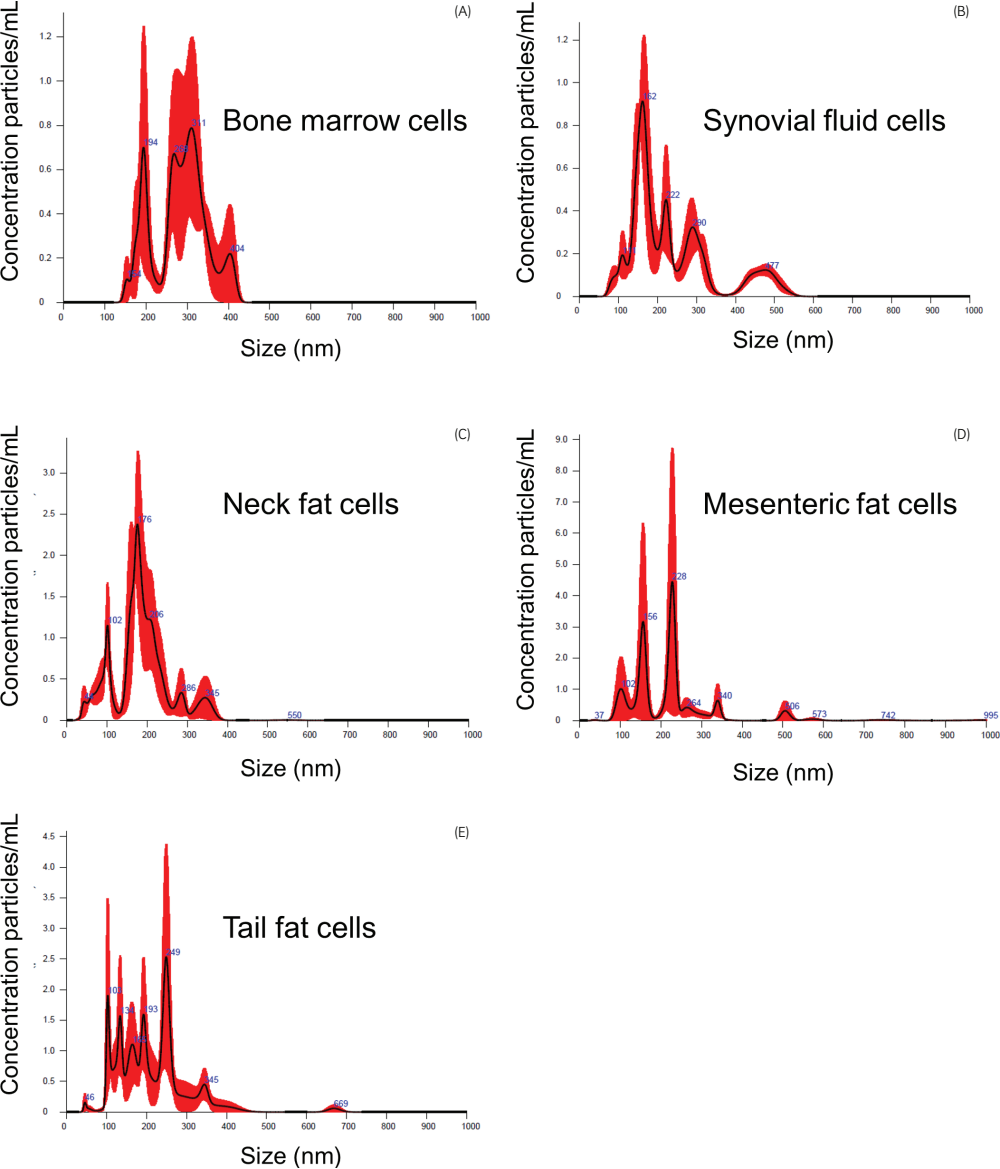
Histogram data from nanoparticle tracking analysis of media from MSCs. Particle sizes are expressed in nm. Particles from adipose cells (C, D, and E) seemed to vary more in size. Mesenteric fat (D) seemed to be more dispersed in comparison with the sizes of other adipose fat cells

NanoSight analysis provided information on particle size. The histogram distribution of obtained data revealed that particles derived from BM‐MSCs ranged in size from 154 to 404 nm (Figure 1A), while particles produced by SF‐MSCs ranged from 111 to 477 nm (Figure 1B). The range of particles produced by adipose‐derived MSCs appeared to be more heterogeneous (Figure 1C‐E). Histograms revealed that: NF‐MSCs derived EVs (Figure 1C) sizes ranged from 40 to 550 nm; particles from mesenteric fat MSCs ranged from 37 to 742 nm (Figure 1D); tail fat MSCs produced particles ranged from 46 to 669 nm (Figure 1E).

### 3.2 Transmission electron microscopic assessment of the morphology of extracellular vesicles

**FIGURE 2:**
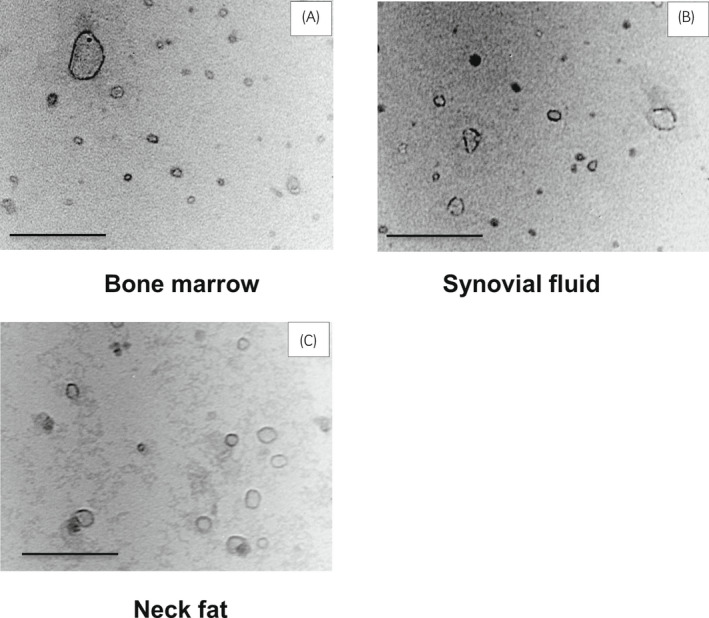
TEM images of isolated particles (EVs)‐derived from BM, SF, and NF cell media. Particles displayed a round‐like shape with a visible membrane. (A) A larger extracellular vesicle observed among the other small extracellular vesicles. (B) and (C) Rounded extracellular vesicles and different particle sizes were observed. Images were acquired at 250 000× magnification. Bar = 0.125 µm

To confirm whether the particles isolated from the derived MSC cultures were indeed EVs, the collected particle suspensions were observed by TEM at a 250 000× magnification. Particles described as “cup‐shaped” were evident for the EVs from three types of MSC cultures (BM, SF, and NF adipose cells) (Figure 2). EVs derived from BM‐MSCs displayed small, medium, and large particle sizes (Figure 2A). EVs from SF‐MSCs were heterogeneous (Figure 2B), while those from NF‐MSCs were more homogeneous (Figure 2C).

**FIGURE 3:**
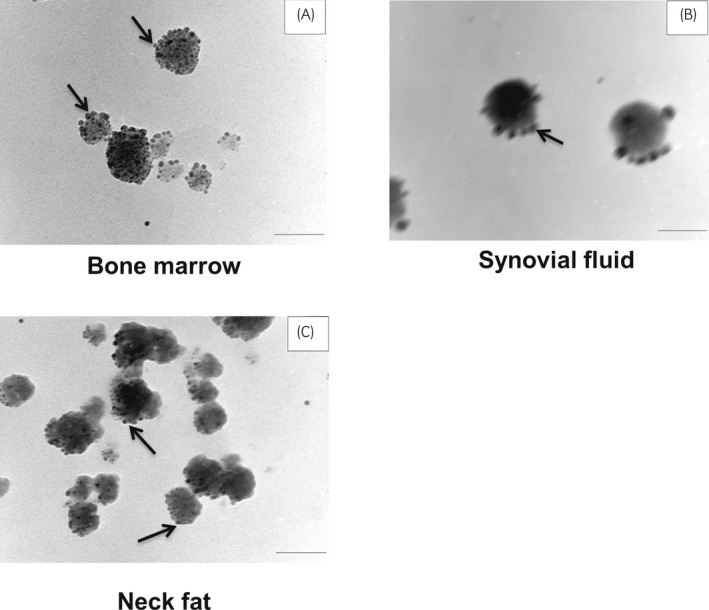
TEM images show immunogold staining with CD9. Arrows in panels A, B, and C indicate darker dots that represent the staining of marker CD9 surrounding the particles (EVs). Scale bar = 0.1 µm

CD9 immunostaining defined the EV identity of these particles from the MSC‐derived media. When stained for CD9, darker dots (ie gold particles) could be observed on the surface of these vesicles (Figure 3), confirming the expression of such markers and their EV designation, as previously reported for equines.^12^

### 3.3 Evaluation of extracellular vesicles in a pro‐inflammatory assay in chondrocytes in vitro

**FIGURE 4:**
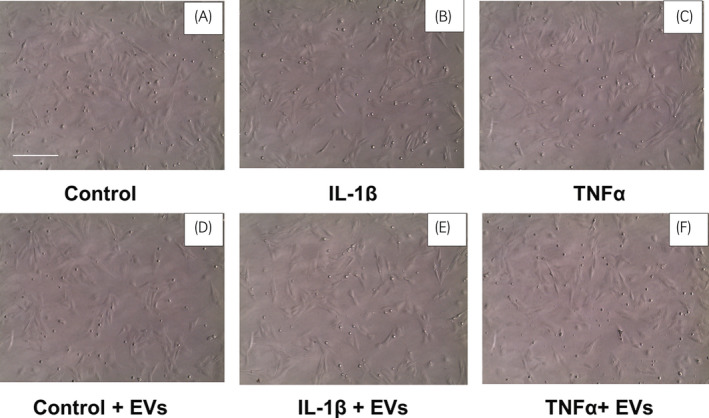
Equine chondrocytes after treatment for 24 h with pro‐inflammatory cytokines. (A) Chondrocytes with control medium, (B) and (C) chondrocytes treated with cytokines, (D) chondrocytes treated with control medium supplemented with EVs, (E) and (F) chondrocytes treated with cytokines and EVs. Chondrocytes display rounded morphology. Images are representative of chondrocytes treated with BM‐MSCs cell‐derived EVs. Images were acquired at 10× magnification. Scale bar = 250 μm

On confirming the characteristics of the various EVs, their anti‐inflammatory potential^5^ in pre‐inflamed chondrocytes was evaluated. Assays using equine chondrocytes, which are a cell population affected during OA,^2^ were performed. Chondrocytes were treated with either pro‐inflammatory cytokines alone (IL‐1 or TNF‐α) or with the same cytokines and isolated EVs. The goal for these treatments was to evaluate whether EVs from BM‐ and SF‐MSCs could counteract or reduce the effect of pro‐inflammatory cytokines on chondrocytes. For this purpose, the cells were starved to eliminate the effect of FBS. To assess the effect of starvation on chondrocytes, cells were observed under the microscope to assess cell viability. The starved chondrocytes retained their typical polymorphic morphology, as shown with control cells (Figure 4A). Starved chondrocytes were incubated for 24 hours with IL‐1β and TNF‐α pro‐inflammatory cytokines, and nuclei were counted using Hoechst 33342. The comparison of cell counts using a one‐way ANOVA revealed no differences among treatments (Figure S1) (*P* > .05). Subsequently, starved chondrocytes were treated for 24 hours with pro‐inflammatory cytokines alone or alongside EVs. Cell morphology did not differ among the treatments (Figure 4).

### 3.4 Extracellular vesicles derived from bone marrow mesenchymal stem cell medium

**FIGURE 5:**
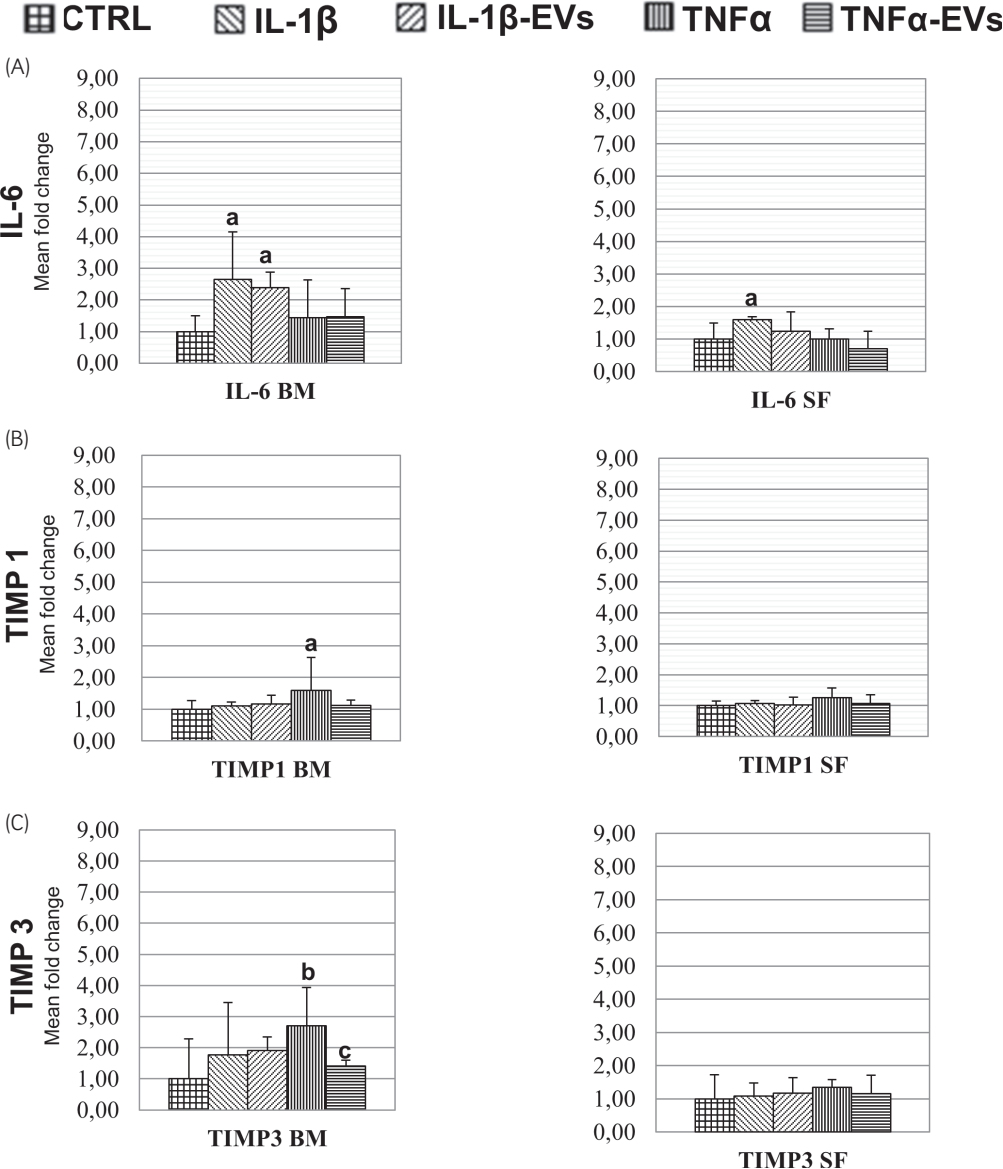
Changes in expressions of genes for IL‐6 and TIMP family members according to treatments. Fold increase or decrease compared to the control (CTRL) from each respective treatment. Gene expression of IL‐6 (A), TIMP 1 (B), and TIMP3 (C). For each panel, the average (n = 3) of each treatment is shown. Differences in letters show statistical significance at *P* < .05

RNA was extracted from chondrocytes and gene expression was evaluated by real‐time PCR. Gene expression levels were compared between the cytokine‐only treatment and untreated control and the respective cytokine + EV treatment against both the control and cytokine‐treated cells. Statistically significant differences were found in the expression of the IL‐6 gene when comparing control (CTRL) with IL‐1β treated or IL‐1β + EVs treated cells (*P* < .001) (Figure 5A). In the analysis of the expression of TIMPs, significant differences were found only in the expression of TIMP‐1 and TIMP‐3 when EVs were added. The expression of TIMP‐1 was increased when cells were treated with TNF‐α, which reverted to control levels when EVs were added (*P* = .001) (Figure 5B). In the case of TIMP3, a similar behaviour was evident when TNF‐α was added in combination with EVs (*P* = .005) (Figure 5C).

**FIGURE 6:**
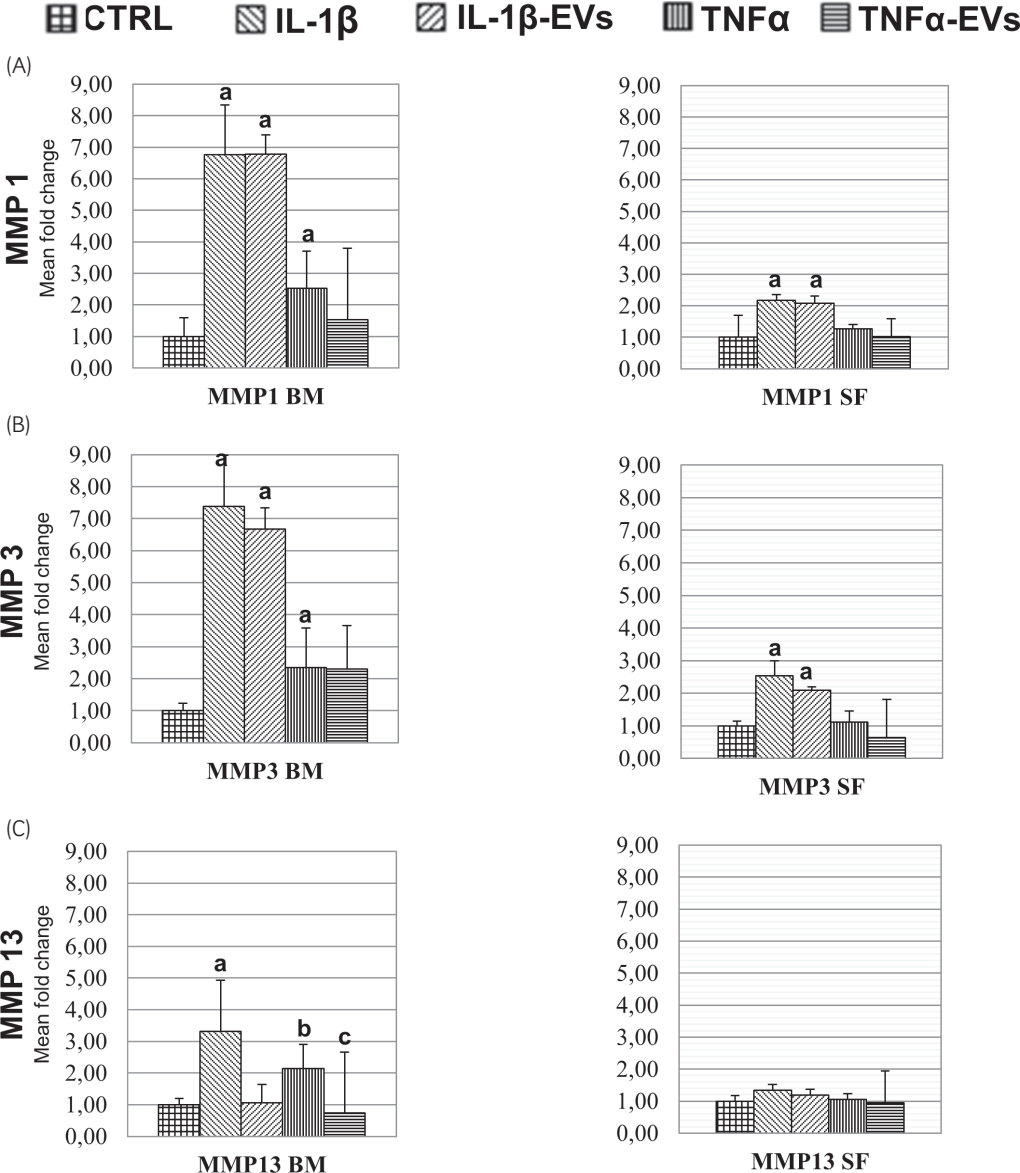
Changes in gene expressions for MMP family members according to treatments. Fold increase or decrease compared to the control (CTRL) from each respective treatment. A, B and C show graphs of the gene expression of MMP1, MMP3, and MMP13 respectively. For each panel, the average (n = 3) of each treatment is shown. Differences in letters show significance at *P* < .05

When using BM‐MSC derived EVs and IL1‐β as an inflammatory signal, MMP1 gene expression did not change with the addition of EVs (Figure 6A). However, when analysing the effect of TNFα, the addition of EVs was able to counteract the effect of the inflammatory cytokine (Figure 6A).

MMP3 and 13 were significantly different in all the comparisons of CTRLs against IL‐1β treated cells, with a difference that was particularly marked for MMP3 (7‐fold change, *P* < .001) (Figure 6B). Whereas MMP13, when comparing CTRL and IL‐1β, displayed intermediate results (3‐fold change) (Figure 6C). Interestingly, a decrease in MMP13 expression to control levels was observed when chondrocytes were treated with EVs along with IL‐1β. A similar pattern was observed after TNF‐α treatment (Figure 6C).

### 3.5 Extracellular vesicles derived from synovial fluid mesenchymal stem cell medium

The effects of EVs derived from SF‐MSCs were also evaluated. However, significant differences were observed only between IL‐1β treatment and CTRL for IL‐6 (*P* = .014); however, no differences were observed between control and IL‐1β and EVs co‐treated cells (Figure 5A).

The expression of MMP1 was significantly different in the case of CTRL compared to the IL‐1β cytokine and CTRL versus IL‐1β‐EVs (both *P* < .001) (Figure 6A). For the MMP3 gene, significant differences were observed between CTRL and IL‐1β, and CTRL and IL‐1β –EVs (both *P* < .001) (Figure 6B).

## 4 DISCUSSION

Extracellular vesicles in culture media derived from different equine MSCs were identified. The recovered EVs were added to equine chondrocytes that were previously subjected to a pro‐inflammatory treatment, and the effects during inflammation of EVs derived from BM‐ and SF‐MSCs were evaluated.

In this study, the number of particles isolated per mL derived from adipose‐derived MSCs was marginally higher than that previously reported for equine adipose cells.^12^ For example, the EVs isolated from adipose cells obtained from similar regions, such as the tail base, were 5.85 × 10^10^ particles/mL, while Klymiuk et al^12^ obtained 9.6 × 10^8^ particles/mL from subcutaneous adipose tissue from the dorsal gluteal muscle region. However, a comparison of the amount of particles obtained per mL from different studies is difficult due to lack of standardised collection protocols and also variations in number of cells seeded in vitro. There were no significant differences in size among the different types of derived MSCs. Particles derived from adipose cells from equine in this study ranged from an average of 151 nm for neck fat to an average of 217 nm for mesenteric fat cells. The sizes of the adipose tissue‐MSC particles observed were similar to that reported previously,^12^ with sizes ranging from 30 to 353 nm. EVs isolated from SF‐MSCs in this study had an average size of 180.2 ± 64.4 nm, which is consistent with the size range (20‐200 nm) found by Boere et al from the SF‐MSC EVs of healthy equines.^20^ In this study, the average size of particles derived from equine BM‐MSCs was 235 nm. EVs derived from human BM‐MSCs have a reported average size of 218 nm^21^ and a maximum size of 241 nm. However, differences in the size of EVs have been observed in the same type of cells after using different isolation protocols, including ultrafiltration, ultracentrifugation, precipitation, and size exclusion chromatography.^12^ TEM determination of particle size diameter may also be influential.^7^, ^22^ However, in this study, all the particles derived from the five different MSC types of derived EVs displayed a mixed population that can be observed via nanoparticle tracking analysis. Our findings echoed those of a previous study involving MSCs.^7^ Our TEM analysis also detected CD9 expression in round‐shaped particles, as similarly reported in equines.^12^ We also tried to detect CD63 expression in the EVs we isolated. However, we were not able to detect CD63 positive staining in our equine EVs in TEM, much like Klymiuk et al reported.^12^ It seems that in equine CD63 expression is observed in cell cultures but not in isolated EVs via immunostaining. CD81 has been reported to be expressed in equine EVs.^12^ However, we decided not to evaluate it since our scope was to confirm the presence of EVs from MSCs media, not to perform a full characterisation of the isolated EVs such as the one previously reported by Klymiuk et al.^12^ Moreover, the lack of available antibodies that cross‐react with equine's EVs hampers research since most of them are developed for other species (mainly human or mouse) use.

The effects of BM and SF‐MSC EVs on chondrocytes were analysed using an inflammation assay. The exposure of chondrocytes to pro‐inflammatory cytokines (IL‐1β and TNFα) has been related to OA pathology.^23^ IL‐1β stimulates IL‐6 gene expression in chondrocytes. Such effects have been studied in OA pathology, since in the presence of IL‐1β and TNFα, IL‐6 activates the immune system and produces an inflammatory response.^23^ Our results coincide with another in vitro study, wherein upregulation of the IL‐6 gene was observed after treatment with IL‐1β in equine chondrocytes.^24^ The findings indicate a direct relationship between IL‐1β and IL‐6 expression in chondrocytes, further indicating that there is the production of IL‐6 in exposed tissues in the presence of IL‐1β.^23^

When EVs derived from BM‐ and SF‐MSCs were added as co‐treatments in chondrocytes, a tendency of reduced IL‐6 expression was observed, although this was not statistically significant. A reduction in cytokines has been reported in stimulated chondrocytes treated with BM‐MSC derived EVs in vitro.^9^

Chondrocytes treated with TNF‐α showed a significant increase in TIMP1 gene expression, which reverted when BM‐MSC derived EVs were added. This could correspond to the regulation of TIMP1, an inhibitor of metalloproteinases.^25^ TIMPs are important modulators of extracellular matrix (ECM) remodelling, and the expression of TIMPs after inflammatory cytokine exposure seems to be generally reduced. Remodelling of the extracellular matrix has also been observed in healthy patients.^25^ Therefore, TIMP3 increase in cells has also been related to the stimulation of cell growth and proliferation.^25^

MMPs should be upregulated during inflammation. Thus, when cells are exposed to IL‐1β and TNFα, upregulation has been reported.^23^ When EVs derived from BM‐ and SF‐MSCs were added, MMP1 expression was slightly reduced in the TNFα treatments. MMP1 expression is usually higher in OA.^23^ MMP1 degrades collagen, reduces elasticity, and alters cartilage. MMP1 is present during the early phases of OA disease in horses.^26^ Therefore, EVs may help to reduce the impact of MMP1 by re‐establishing the cartilage extracellular matrix and tissue regeneration^5^ during the early stages of OA.^26^ MMP3 and MMP13 expressions were increased during cytokine treatment, similar to other studies.^18^, ^23^ When BM‐MSC derived EVs were added, a significant reduction in MMP13 was observed. MMP3 has been related to the pathological and degenerative state of OA. EVs may act as immunomodulators and might influence cells in regenerative programmes.^5^ Accordingly, for athletic horses, the expectation is that further loss of morphological changes in the cartilage could be prevented.

These beneficial results from BM‐MSC derived EVs point to a possible effect on extracellular matrix remodelling during inflammation by altering the expression of one of the many components of this system. TNFα effect was reduced after adding EVs derived from BM‐MSCs to MMP13 and SF to MMP1 expression. These results might have implications for the improvement and preservation of the ECM.^27^

Extracellular vesicles isolated from BM‐MSCs caused a significant reduction in MMP13 gene expression in the in vitro inflammation model. Such an MMP13 reduction has been previously demonstrated in cartilage degradation in OA using a conditioned medium from MSCs.^10^, ^27^ Since EVs are released from cells in culture media,^6^ BM‐MSC EVs could be responsible for the effects of the conditioned medium.

Synovial fluid in joints provides growth factors and nutrients for chondrogenesis of articular cartilage,^3^ and it is known that SF cells synthesise collagen.^28^ However, the EVs derived from SF‐MSCs were not as efficient as EVs derived from BM‐MSCs in modulating the expressions of genes related to inflammation and ECM degradation. EVs from synovial membrane cells reportedly have less therapeutic value than EVs derived from induced pluripotent MSCs^29^ using the same amount of EVs as treatments for OA in mice. A stronger effect of EVs derived from synovial structures may be obtained in OA by increasing their amount. Therefore, further studies are required to evaluate the potential of these compounds.

Considering all the data presented, the effect of EVs in our in vitro inflammation assay seems to point to different effects according to the origins of the EVs. EVs isolated from specific tissues may affect some genes involved in the inflammatory process. Such behaviour indicates differences in the content of EVs according to the tissue of origin and, therefore, consistent differences in their performances during inflammation. The choice of a single cell type to produce and isolate EVs might result in poorer outcomes in treatment in vivo.

## 5 CONCLUSION

Extracellular vesicles isolated from MSCs derived from BM, adipose, and SF media were analysed in vitro. The average amount and size of EVs did not differ according to their origin, but different size distributions were detected. However, statistical differences were observed between the BM‐ and SF‐MSC derived EVs concerning their effect on gene expressions during inflammation. BM‐MSC EVs seem to have a promising effect on MMPs, mainly MMP13 gene expression. Analysis of the content of EVs (more specifically, RNAs or selected growth factors) might help to understand their effects. Little is known about EV content and its effectiveness as a treatment to improve inflammation in equines. In this context, our findings indicate a novel approach with in vitro data that might help productively adapt a strategy to the in vivo use of MSCs or their derived products in equine leg pathologies.

## ETHICAL ANIMAL RESEARCH

Research ethics committee oversight is not currently required by this journal: the study was performed on material obtained from an abattoir.

## INFORMED CONSENT

Not applicable.

## CONFLICT OF INTERESTS

No competing interests have been declared.

## AUTHOR CONTRIBUTIONS

M. Arévalo‐Turrubiarte was responsible for conceptualisation, investigation, formal analysis, writing ‐ original draft and writing‐review and editing. M. Baratta was responsible for formal analysis, supervision and writing‐original draft. G. Ponti was responsible for investigation, methodology and analysis. E. Chiaradia was responsible for methodology. E. Martignani was responsible for conceptualisation, formal analysis, supervision, writing‐original draft and writing‐review and editing. All authors gave their approval for the final manuscript.

## Supporting information

Fig S1

## Data Availability

The data that support the findings of this study are available from the corresponding author upon reasonable request.
