# Supplementary material for: Extracellular vesicles from equine mesenchymal stem cells decrease inflammation markers in chondrocytes in vitro
Source: Equine Vet J. 2021 Nov 24;54(6):1133–43. doi: 10.1111/evj.13537 (PMC9787580; doi:10.1111/evj.13537)

Equine Veterinary Journal BEVA

Figure S1: Analysis of pro-inflammatory cytokines on chondrocytes proliferation. Cell counts shown are the average of three technical replicates (n=3) ± SD. No statistical difference was found among treatments.

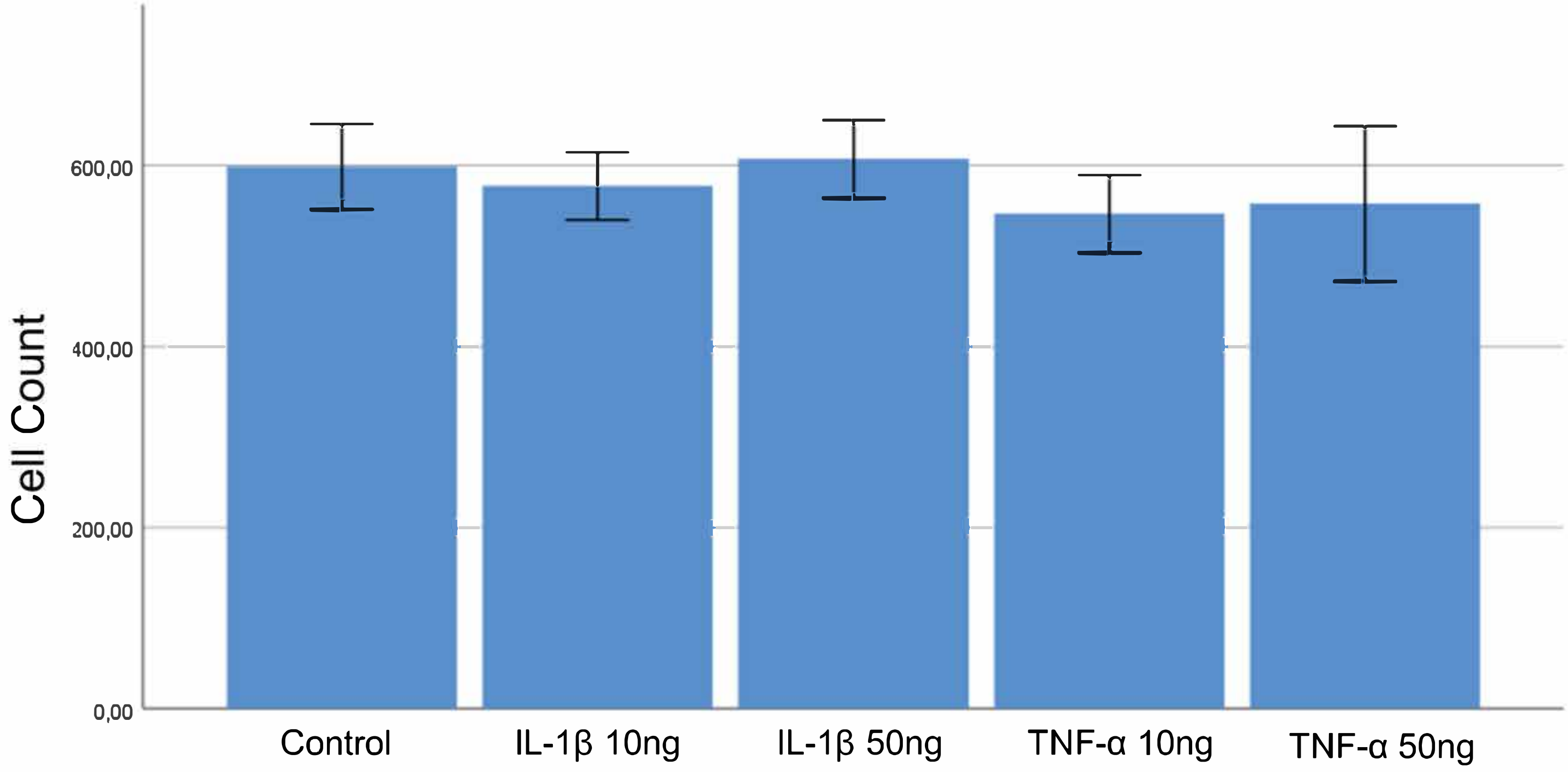

Supplement: Supplementary file 1 — Fig S1 [file EVJ-54-1133-s001.pdf]
